# Early-onset phenotype in a patient with an intermediate allele and a large SCA1 expansion: a case report

**DOI:** 10.1186/s12883-024-03846-2

**Published:** 2024-09-17

**Authors:** Guillaume Baille, Nicolas Geoffre, Anna Wissocq, Pauline Planté-Bordeneuve, Eugénie Mutez, Vincent Huin

**Affiliations:** 1 Delafontaine Hospital Center, Department of Neurology, Saint-Denis, F93200 France; 2 Department of Toxicology and Genopathies, UF Neurobiology, CHU Lille, Lille, F-59000 France; 3 Department of Neurology and Movement disorders, CHU Lille, Lille, F-59000 France; 4 Univ. Lille, CHU Lille, U1172 - LilNCog - Lille Neuroscience & Cognition, Inserm, Lille, F-59000 France; 5 Inserm UMRS1172, ‘Alzheimer & Tauopathies’, Bâtiment Biserte, Place de Verdun, Lille Cedex, 59045 France

**Keywords:** SCA1, Cerebellar ataxia, Spinocerebellar degenerations, Phenotype, DNA repeat expansion, Polyglutamine disease, Case report

## Abstract

**Background:**

Spinocerebellar ataxia type 1, is a rare neurodegenerative disorder with autosomal dominant inheritance belonging to the polyglutamine diseases. The diagnosis of this disease requires genetic testing that may also include the search for CAT interruption of the CAG repeat tract.

**Case presentation:**

One 23-years-old patient suffers from a severe ataxia, with early-onset and rapid progression of the disease. His father might have been affected, but no molecular confirmation has been performed. The genetic results were negative for the Friedreich’s ataxia, spinocerebellar ataxia type 2, 3, 6, 7 and 17. The numbers of CAG repeats in the *ATXN1* gene was assessed by fluorescent PCR, tripled-primed PCR and enzymatic digestion for the search of sequence interruption in the CAG repeats. The patient carried one pathogenic allele of 61 CAG and one intermediate allele of 37 CAG in the *ATXN1* gene. Both alleles were uninterrupted.

**Conclusions:**

We report a rare case of spinocerebellar ataxia type 1 with an intermediate allele and a large SCA1 expansion. The determination of the absence of CAT interruption brought crucial information concerning this molecular diagnosis, the prediction of the disease and had practical consequences for genetic counseling.

**Supplementary Information:**

The online version contains supplementary material available at 10.1186/s12883-024-03846-2.

## Background

Spinocerebellar ataxia type 1 (SCA1), is a rare neurodegenerative disorder with autosomal dominant inheritance belonging to the polyglutamine diseases. It is caused by a CAG repeat expansion, sometimes interrupted by CAT repeats, in *ATXN1* gene on chromosome 6p22 [1]. The disease usually begins in the fourth decade of life and is characterized by progressive cerebellar ataxia, dysarthria and evolution to bulbar dysfunction. The disease progresses slowly with muscle atrophy, decreased deep tendon reflexes, loss of proprioception, and cognitive impairment. Other rare neurological impairments may include upper motor neuron signs and movement disorders such as chorea or dystonia [2]. The molecular diagnosis of SCA1 is based on fluorescence-labeled PCR and fragment length analysis to determine the number of the CAG repeats. However, there is sometimes an interest in looking for the presence of CAT trinucleotides that interrupt the CAG repeat tract. Indeed, allele between 36 and 44 repeats measured by fluorescent PCR can be either normal, intermediate (or mutable normal allele), or pathogenic depending on the presence or not of CAT interruptions [1] (Supplementary Table S1). Intermediate alleles have not been associated with symptoms in SCA1, but there is growing evidence that intermediate alleles in polyglutamine diseases may have a significant effect on clinical outcomes [3].

We describe the case of a patient with a severe and early-onset ataxia, which progressed rapidly, associated with one pathogenic expansion on one allele and an intermediate allele.

## Case presentation

A 23-year-old patient of Tamil origin came to our outpatient clinic because of walking difficulties. Since the age of 15, he suffered from slowly progressive action tremor, clumsiness and gait trouble. At the age of 19, he could not run anymore. For one year, he has started to use crutches, as he fell several times. The use of a wheelchair has now been proposed to the patient. He had no other medical history. His father died at the age of 40 during a civil war, but he was known to suffer from tremor and poor balance. There is no history of gait disturbance in his mother and two older siblings. Molecular testing was not previously performed on the possibly affected father, nor on unaffected family members. The clinical examination highlighted a severe cerebellar ataxia with hypotonia, hyperreflexia with extensor plantar reflex and a marked dysarthria. The Scale for the Assessment and Rating of Ataxia score [4] was 25/40. Neither dysautonomia nor parkinsonism were found and the extra-neurological examination was normal. There was no evident cognitive dysfunction. Brain MRI revealed a severe isolated cerebellar atrophy (Supplementary Fig. S1). The vitamin E and Alpha-fetoprotein levels were normal, as well as the fundus. The electromyography showed a mild axonal length-dependent neuropathy. A blood sample was then addressed to our laboratory for the molecular diagnosis of cerebellar ataxia.

**Fig. 1:**
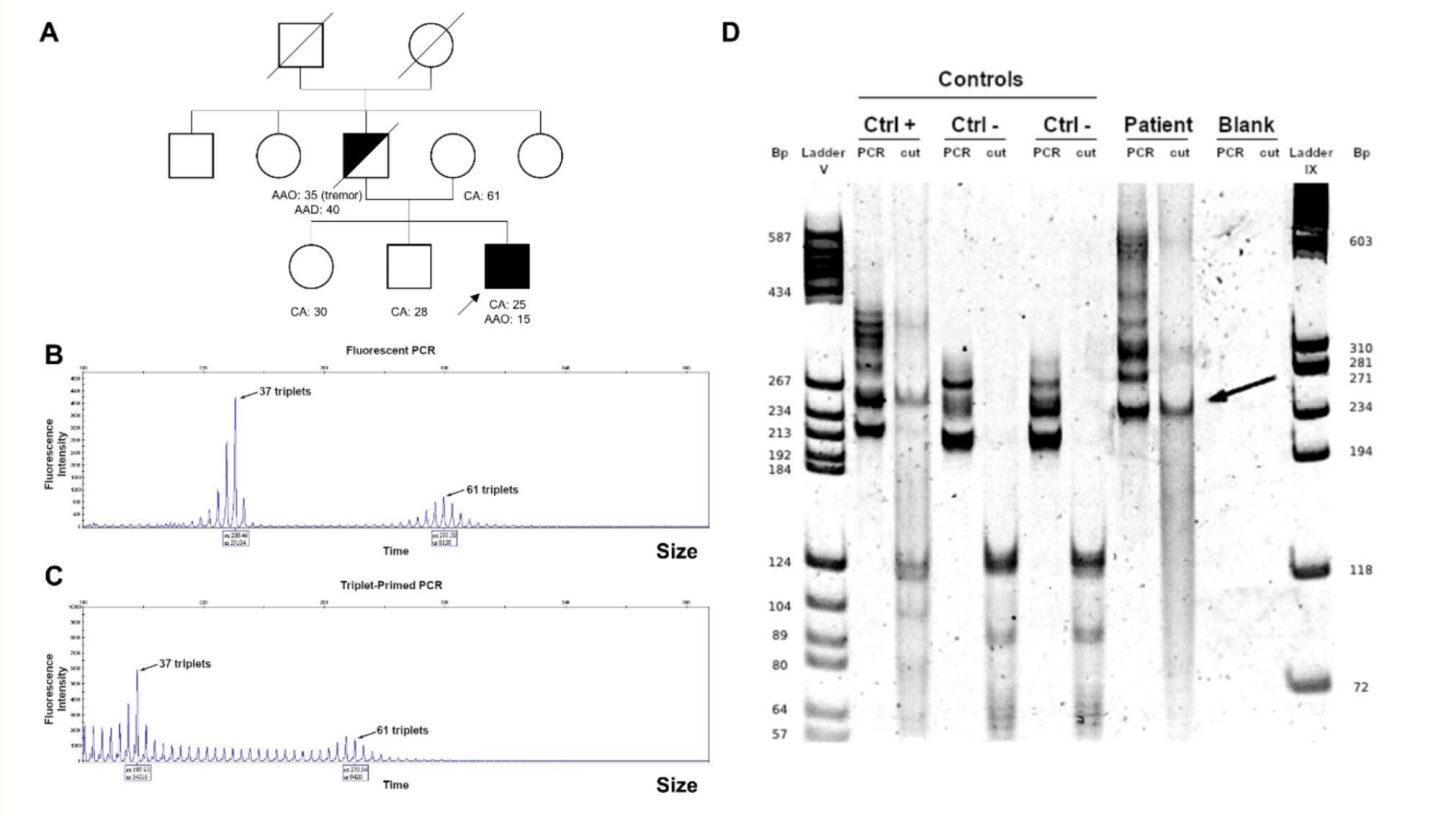
Pedigree and molecular analyses of the SCA1 locus **(A)** Pedigrees of the family. Circles denote women. Squares denote men. The proband is indicated by an arrow. Filled black symbols denote clinically affected members. Half-blackened symbols indicate individuals affected by history. Open symbols indicate unaffected individuals. A forward slash indicates deceased individuals. CA: Current age; AAD: Age at death; AAO: Age at onset of disease. Patient’s capillary electropherograms at SCA1 locus using fluorescent PCR **(B)** and TP-PCR **(C)**. These analyses display two alleles, one at uncertain pathogenicity (37 triplets) and one pathogenic allele (61 triplets). **(D)** Sequence interruption analysis. The 230 bp band (37 triplets) in the patient sample remains present after enzymatic digestion (arrow) and no cleavage products around 90–130 bp are seen. bp: base pairs; Ctrl +: positive control; Ctrl -: negative control

The genetic results were negative for the Friedreich’s ataxia, SCA2, 3, 6, 7 and 17. We evidenced two expanded alleles of *ATXN1* gene, one pathogenic allele of 61 repeats and a second allele of 37 repeats of unknown significance (Fig. 1.A-B). Between 36 and 38 repeat, such allele can be normal or unstable depending on the presence of CAT interruption (Supplementary Table S1). To assess the stability and the impact for the genetic counseling of this second allele, we performed enzymatic digestion of PCR products with SfaNI. We showed that both alleles are uninterrupted (Fig. 1.C). The shorter allele of 37 CAG repeats was classed as an intermediate allele (or mutable normal allele). Such intermediate allele is not associated with symptoms but is unstable and can expand into the abnormal range on transmission to offspring.

## Discussion and conclusions

In this case, the determination of the absence of CAT interruption brought crucial information and lead to the unexpected discovery of an unstable allele with intermediate size. This has practical consequences for genetic counseling, which is proving to be quite complex in this family. Indeed, all the proband’s first-degree relatives, and especially his sibling has high risk to develop the disease later in life, but also to have an intermediate allele and to transmit to their offspring an allele whose CAG expansion size would move it into the full-penetrance zone.

This case report raised the question of the putative contribution of the intermediate allele to the clinical phenotype in SCA1. It has been reported for other polyglutamine diseases (i.e. SCA3, SCA6, SCA17 and DRPLA) that homozygotes patients show earlier onset and more severe manifestations than heterozygotes [5–8]. In Huntington disease, the rare homozygous patients have similar age at onset than the heterozygotes, but their disease progression may be faster [9]. Moreover, homozygous alleles of intermediate size in SCA2 and SCA6 have been reported with the diseases [10, 11].

On the contrary, the few studies reporting patients with biallelic SCA1 expansions do not support a more severe phenotype in these patients [12, 13] (Table 1). However, our study has the advantage of providing more clinical evidences and a more detailed molecular characterization. This included the search for CAT trinucleotides interruptions in alleles with 39–44 repeats which, if present, can correspond to a normal allele. Two of the five previously reported patients with biallelic SCA1 expansions might thus not carry two pathogenic alleles, but rather have only one pathogenic allele and one normal interrupted allele [12, 13].

**Table 1:** Clinical and genetic characteristics of SCA1 patients with biallelic expansions and our patient

		Goldtarb et al., 1996				Sharma et al., 2022			Present study		
**Clinical data**											
	Status	Asymptomatic	Symptomatic	Symptomatic		Symptomatic	Symptomatic		Symptomatic		
	Age at onset	nc	22	32		na	na		15		
	Other clinical data	Age at examination is not provided	Functional stage 2 (loss of domestic skills)	Functional stage 3 (loss of self-care skills)		na	na		Cerebellar ataxia, pyramidal syndrome, dysarthria, SARA-score: 25/40 at age 23		
**Molecular screening**											
	Flanking PCR	+	+	+		+	+			+	
	Triplet-primed PCR	-	-	-		-	-			+	
	Interruption analysis	-	-	-		-	-			+	
**Results of the molecular analysis**											
	**Larger allele**										
	Size	54	56	50		48	54			61*	
	Interpretation	Pathogenic with full penetrance	Pathogenic with full penetrance	Pathogenic with full penetrance		Pathogenic with full penetrance	Pathogenic with full penetrance			Pathogenic with full penetrance	
	**Smaller allele**										
	Size	45	48	44		48	43			37*	
	Interpretation	Pathogenic with full penetrance	Pathogenic with full penetrance	Normal interrupted allele OR pathogenic with full penetrance?		Pathogenic with full penetrance	Normal interrupted allele OR pathogenic with full penetrance?			Intermediate allele	

na: not available; nc: not concerned; * uninterrupted allele; SARA-score: Scale for the Assessment and Rating of Ataxia score

In SCA1, as in numerous polyglutamine diseases, the age at onset inversely correlates with the size of the pathogenic expansion [14] and, more specifically with the longer uninterrupted CAG stretch [15]. The progression of the disease in our patient is also very rapid. This may be explained again by the size of the pathogenic expansion which is associated with faster progression of the SARA score [16]. Similarly, the rare, infantile- or juvenile-onset SCA1 have been reported with a very severe disease and a rapid progression [17]. A large European study of 317 SCA1 patients from the EUROSCA registry reported a correlation between triplet repeats in the pathogenic alleles and a younger age of onset. However, the authors also reported that the number of triplets in the non-pathogenic alleles inversely correlated with a younger age of onset in SCA1 patients [18]. One limitation of our study is that we cannot exclude the role of other genetic determinants, or putative environmental exposures that might have influenced the course of the disease in our patient. For example, in a recent work, Kacher et al., reported that somatic instability of the CAG repeats correlates with the clinical progression for SCA1 individuals [19]. Alleles of intermediate size without CAT interruption in *ATXN1* gene are mutable and display somatic instability too [1]. The somatic instability of both alleles in our patient may thus have been implicated in modifying age of disease onset, progression or gravity of the phenotype. It will be necessary to add other descriptions to confirm or refute our results.

In conclusion, we report a rare case carrying a full penetrant allele of 61 CAGs and an intermediate allele of 37 uninterrupted CAG repeats in *ATXN1* gene in a patient with a severe phenotype. The determination of the absence of CAT interruption brought crucial information concerning the prediction of the disease and the genetic counseling of this family.

## Electronic supplementary material

Below is the link to the electronic supplementary material.

Supplementary Material 1

## Data Availability

No datasets were generated or analysed during the current study.

## Abbreviations

ATXN1: Ataxin 1
MRI: Magnetic resonance imaging
SCA: Spinocerebellar ataxia
TP-PCR: Triplet-primed polymerase chain reaction
