# Supplementary material for: Early-onset phenotype in a patient with an intermediate allele and a large SCA1 expansion: a case report
Source: BMC Neurol. 2024 Sep 17;24:348. doi: 10.1186/s12883-024-03846-2 (PMC11406724; doi:10.1186/s12883-024-03846-2)

# SUPPLEMENTARY DATA

## SUPPLEMENTARY METHODS

### Fragment length analysis

Genomic DNA was extracted from patient’s peripheral blood leukocytes using standard procedures. PCR protocols are shown in Supplementary Table S2. PCR products from the fluorescence labeled PCR or from the TP-PCR were loaded on an ABI Prism 3730XL automatic sequencer (Applied Biosystems™, Carlsbad, USA, CA) on a 36-cm capillary with the POP7 polymer type and the 8,5 μl formamide and the GS-600Liz size standard (Bioz Inc, Los Altos, USA, CA). Temperature was set to 60 °C, injection voltage to 2.5 kV, and injection time to 20 seconds. The bioinformatics analysis was performed using the GeneMapper® Software version 6.0 (Applied Biosystems™, Carlsbad, USA, CA) and GeneMarker® software version 2.6 (Softgenetics, State College, USA, PA).

The size of the PCR products on the electropherogram was recorded, then we calculated the size of the CAG/CAT expansions in number of triplets using the fluorescence labeled PCR. The size was calculated by taking into account the last major peak observed on the electropherogram, after deducting the size of the constant regions amplified by the PCR and dividing by 3. The number of CAG/CAT triplets was obtained from the following formulas:

-Fluorescence labeled PCR: (CAG/CAT)ₙ = (X base pairs - 118) / 3

-TP-PCR: (CAG/CAT)ₙ = (X base pairs - 87) / 3

Fluorescence labeled PCR and TP-PCR analyses have a measurement uncertainty of ±1 triplet.

### Sequence interruption analysis

The presence of CAT interruption within the expanded allele of intermediate size was inferred by SfaNI restriction analysis [2]. Briefly, we amplified of the SCA1 locus by fluorescence labeled PCR and performed enzymatic digestion of 10 µl PCR products with SfaNI. The restriction enzyme SfaNI recognizes 5’-GCATC(N)_5_-3’ and cuts then the PCR product if a CAT interruption is present and allows the distinction of large normal (interrupted) alleles and uninterrupted alleles. After enzymatic digestion, 10 µl of PCR products (digested or not) were loaded on a 5% acrylamide gel. We used the DNA Molecular Weight Marker V (Roche) 2 µl, and Marker IX (Boehringer) 2 µl. After electrophoresis, we colored the acrylamide gel with Ethidium bromide.

## Supplementary Table S1: Reference values for interpreting the fluorescent PCR at the SCA1 locus

|  | Normal alleles | | Intermediate / Mutable normal alleles | Full-penetrance alleles | |
| --- | --- | --- | --- | --- | --- |
|  | Always | With (CAT) interruption | Without (CAT) interruption | Without (CAT) interruption | With (CAT) interruption |
| Size (bp) | 136-223 | 226-250 | 226-232 | ≥235 | ≥253 |
| Triplet number | 6-35 | 36-44 | 36-38 | ≥39 | ≥45 |

bp: base pairs.

## Supplementary Table S2: Experimental conditions used for Fluorescence labeled PCR and TP-PCR protocols

***Fluorescence labeled PCR***

| **Reagents**  1. Taq DNA Polymerase recombinant Invitrogen 5U/µl  2. TpTaq (NH_4_)_2_SO_4_ 10X  3. MgCl2 30 mM  4. dNTP 25 mM  5. Primers 1 Μm  6. DMSO 10%  7. gDNA 100 ng | **Primers**  **SCA1-Fluorescence labeled PCR**  F: 6-FAM-CAACATGGGCAGTCTGAG  R: AACTGGAAATGTGGACGTA | **Cycling conditions**  94°C x 4 min  59°C x 2 min  [72°C x 90 sec  94°C x 1 min  59°C x 1 min – Each cycle decreasing by 2,5°C] x 35 cycles  72°C x 10 min  10°C x 15 min |
| --- | --- | --- |

***TP-PCR***

| **Reagents**  1. Taq DNA Polymerase recombinant Invitrogen 5U/µl  2. TpTaq (NH_4_)_2_SO_4_ 10X  3. MgCl2 15 mM  4. dNTP 25 mM  5. P1 primer 1 μM  6. P3 primer 1 μM  7. P4-primer 0.1 μM  8. DMSO 10%  9. Betaine 5 M  10. gDNA 50 ng | **Primers**  **SCA1-TP-PCR**  F:6-FAM-TGGAGGCCTATTCCACTCTG  R: TACGCATCCCAGTTTGAGACG  P4: TACGCATCCCAGTTTGAGACG  TGCTGCTGCTGCTGCT | **Cycling conditions**  97°C x 5 min  [94°C x 30 sec  57°C x 1 min  72°C x 30 sec – Each cycle decreasing by 2,5°C] x 38 cycles  72°C x 10 min  10°C x 15 min |
| --- | --- | --- |

## Supplementary Fig. S1. Brain MRI showing atrophy of the cerebellar hemispheres


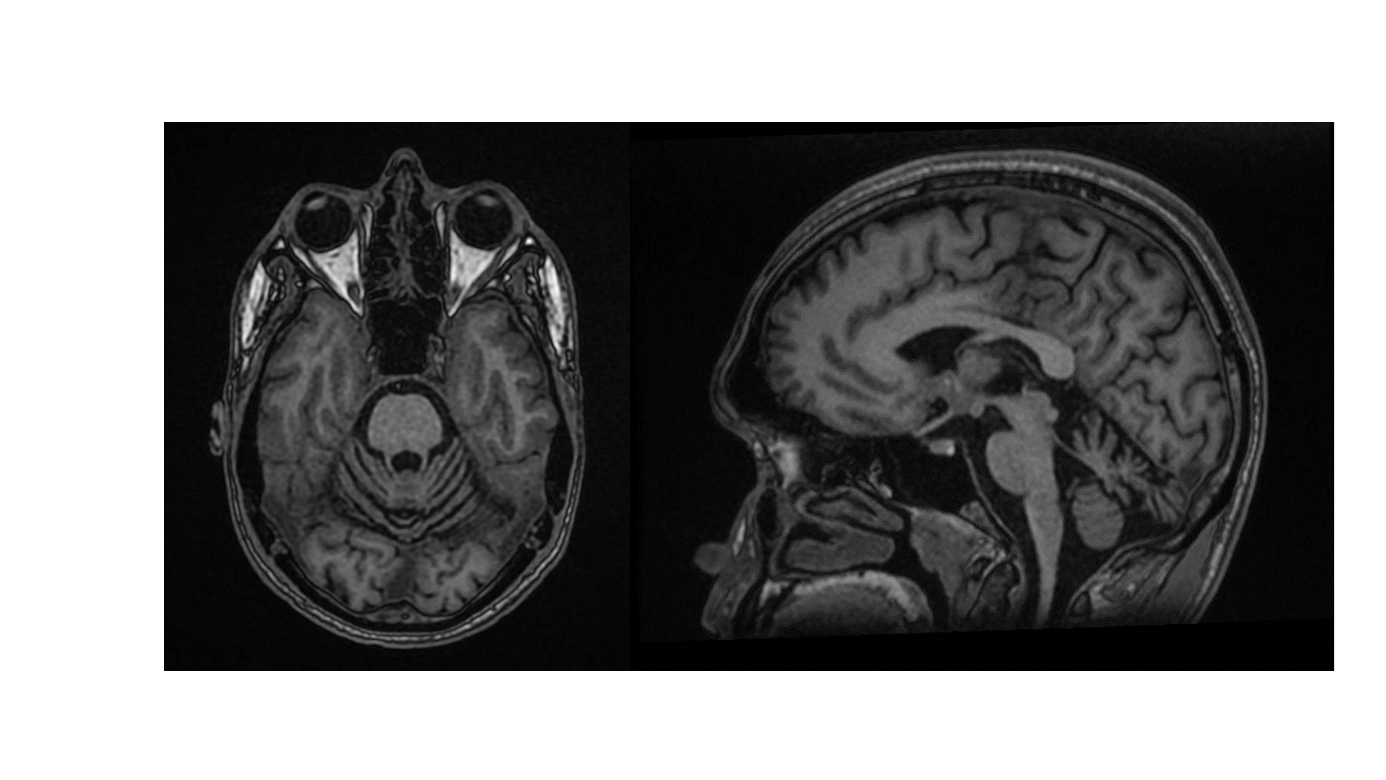

Supplement: Supplementary file 1 — Supplementary Material 1 [file 12883_2024_3846_MOESM1_ESM.docx]
